# Early Effects of Passive Leg-Raising Test, Fluid Challenge, and Norepinephrine on Cerebral Autoregulation and Oxygenation in COVID-19 Critically Ill Patients

**DOI:** 10.3389/fneur.2021.674466

**Published:** 2021-06-16

**Authors:** Chiara Robba, Antonio Messina, Denise Battaglini, Lorenzo Ball, Iole Brunetti, Matteo Bassetti, Daniele R. Giacobbe, Antonio Vena, Nicolo' Patroniti, Maurizio Cecconi, Basil F. Matta, Xiuyun Liu, Patricia R. M. Rocco, Marek Czosnyka, Paolo Pelosi

**Affiliations:** ^1^Department of Surgical Sciences and Integrated Diagnostics (DISC), University of Genoa, Genoa, Italy; ^2^San Martino Policlinico Hospital, Istituto di Ricovero e Cura a Carattere Scientifico for Oncology and Neuroscience, Genoa, Italy; ^3^Humanitas Clinical and Research Center-Istituto di Ricovero e Cura a Carattere Scientifico, Milan, Italy; ^4^Department of Biomedical Sciences, Humanitas University, Milan, Italy; ^5^Department of Health Sciences (DISSAL), University of Genoa, Genoa, Italy; ^6^Infectious Diseases Unit, San Martino Policlinico Hospital, Genoa, Italy; ^7^Neurocritical Care Unit, Addenbrookes Hospital, Cambridge, United Kingdom; ^8^Department of Anesthesiology and Critical Care Medicine, John Hopkins University, Baltimore, MD, United States; ^9^Laboratory of Pulmonary Investigation, Carlos Chagas Filho Institute of Biophysics, Rio de Janeiro, Brazil; ^10^Brain Physics Laboratory, Department of Clinical Neurosciences, University of Cambridge, Cambridge, United Kingdom

**Keywords:** fluid challenge, norepinephrine, passive leg raising test, cerebral oxygenation, cerebral autoregulation

## Abstract

**Background:** Coronavirus disease 2019 (COVID-19) patients are at high risk of neurological complications consequent to several factors including persistent hypotension. There is a paucity of data on the effects of therapeutic interventions designed to optimize systemic hemodynamics on cerebral autoregulation (CA) in this group of patients.

**Methods:** Single-center, observational prospective study conducted at San Martino Policlinico Hospital, Genoa, Italy, from October 1 to December 15, 2020. Mechanically ventilated COVID-19 patients, who had at least one episode of hypotension and received a passive leg raising (PLR) test, were included. They were then treated with fluid challenge (FC) and/or norepinephrine (NE), according to patients' clinical conditions, at different moments. The primary outcome was to assess the early effects of PLR test and of FC and NE [when clinically indicated to maintain adequate mean arterial pressure (MAP)] on CA (CA index) measured by transcranial Doppler (TCD). Secondary outcomes were to evaluate the effects of PLR test, FC, and NE on systemic hemodynamic variables, cerebral oxygenation (rSo_2_), and non-invasive intracranial pressure (nICP).

**Results:** Twenty-three patients were included and underwent PLR test. Of these, 22 patients received FC and 14 were treated with NE. The median age was 62 years (interquartile range = 57–68.5 years), and 78% were male. PLR test led to a low CA index [58% (44–76.3%)]. FC and NE administration resulted in a CA index of 90.8% (74.2–100%) and 100% (100–100%), respectively. After PLR test, nICP based on pulsatility index and nICP based on flow velocity diastolic formula was increased [18.6 (17.7–19.6) vs. 19.3 (18.2–19.8) mm Hg, *p* = 0.009, and 12.9 (8.5–18) vs. 15 (10.5–19.7) mm Hg, *p* = 0.001, respectively]. PLR test, FC, and NE resulted in a significant increase in MAP and rSo_2_.

**Conclusions:** In mechanically ventilated severe COVID-19 patients, PLR test adversely affects CA. An individualized strategy aimed at assessing both the hemodynamic and cerebral needs is warranted in patients at high risk of neurological complications.

## Introduction

Severe hypoxemic respiratory failure is the main reason for intensive care unit (ICU) admission in coronavirus disease 2019 (COVID-19) patients (1–3).

However, COVID-19 is a multisystemic disease (4), with significant implications for the brain (5–7). Different mechanisms related to neurological damage have been proposed, such as a direct viral neurotropism, hypercoagulable state, and systemic complications including hypoxia and hypotension (8). Therefore, it seems logical that in order to protect the brain, with optimized cerebral perfusion and oxygenation, hemodynamic stability should be maintained (6).

The current target of mean arterial pressure (MAP) commonly used in the general ICU population (>65 mm Hg) (7) may not always be sufficient to ensure adequate cerebral perfusion, as the brain might potentially require higher values of MAP to optimize cerebral perfusion pressure (CPP) and maintain cerebral autoregulation (CA) (9, 10), especially in the COVID-19 patients who often present altered cerebrovascular dynamics (5). A commonly used functional hemodynamic test to assess fluid responsiveness is the passive leg raising (PLR) test, which causes a shift of intravascular fluids from the legs to the abdominal compartment. Methods proposed to optimize MAP have different pathophysiological mechanisms and include a quick infusion of a small amount of fluids [the so-called fluid challenge (FC)], which provides an extrinsic increase in intravascular volume, and vasopressors, such as norepinephrine (NE), generally used in fluids non-responders, which increase vascular tone.

As no data are available regarding the effect of PLR test on cerebral function and in particular autoregulation in mechanically ventilated severe COVID-19 patients, we conducted a prospective observational study; the primary outcome was to assess the early effect of PLR test and of FC and/or NE—when clinically indicated—on static CA [CA index measured by transcranial Doppler (TCD)]. Secondary outcomes were to evaluate the effects of PLR test, FC, and NE on systemic hemodynamic variables, cerebral oxygenation [regional cerebral oxygen saturation (rSo_2_)], and non-invasive intracranial pressure (nICP).

## Materials and Methods

A single-center, prospective observational study was conducted at Policlinico San Martino, IRCCS for Oncology and Neuroscience, Genoa, Italy. This study is reported according to the Strengthening the Reporting of Observational Studies in Epidemiology statement guidelines for observational cohort studies (Supplementary Material 1) (11). The local ethical review board approved the protocol (Comitato Etico Regione Liguria, protocol n. CER Liguria: 23/2020). Mechanically ventilated patients admitted to ICU during the second wave of COVID-19 pandemic (from the October 1 to December 15, 2020) were included. COVID-19 patients were defined with a confirmed SARS-CoV-2 (severe acute respiratory syndrome coronavirus 2) polymerase chain reaction using nasopharyngeal swab or bronchoalveolar lavage. Inclusion criteria were (1) ≥18 years old; (2) mechanically ventilated severe COVID-19 patients requiring a PLR test as well as FC and/or NE administration, according to the indications of the attending physician, during the occurrence of hypotension [defined as MAP ≤65 mm Hg and/or systolic blood pressure (SBP) ≤90 mm Hg]; and (3) patients undergoing multimodal neuromonitoring [including cerebral oxygenation using near-infrared spectroscopy (NIRS) and TCD].

Exclusion criteria were (1) patients with a limited acoustic window for TCD assessment, which might have led to a non-precise measurement of the cerebral flow velocities; and (2) patients with known neurological conditions before or during ICU admission, which might have impaired CA (stroke, trauma, intracerebral masses, etc.).

### Data Collection

Data were reviewed and collected by physicians trained in critical care patients' electronic medical records. Baseline characteristics, including demographic and clinical data, were collected at ICU admission. Patients' data have been partially previously presented Robba et al. (12). Data collection included age, gender, Sequential Organ Failure Assessment, body mass index, comorbidities (hypertension, diabetes mellitus, chronic kidney injury, chronic respiratory disease, previous neurological disease, liver failure, chronic cardiac disease), laboratory parameters [blood test, d-dimer, C-reactive protein, procalcitonin, creatinine, hemoglobin (Hb)], and ventilatory parameters [tidal volume (V_T_), fraction of inspired oxygen (Fio_2_), respiratory rate (RR), positive end-expiratory pressure (PEEP), plateau pressure (Pplat), and respiratory system compliance].

### General Management in ICU

Patients were sedated with a combination of propofol, midazolam, and fentanyl and mechanically ventilated using pressure-controlled ventilation, aimed at maintaining Pplat <28 cm H_2_O, using a V_T_ of 4–8 mL/kg of predicted body weight. Fio_2_ and PEEP were titrated in order to achieve peripheral saturation of oxygen (Spo_2_) 88–92%, and RR was set to maintain arterial partial pressure of carbon dioxide (Paco_2_) = 35–45 mm Hg. Permissive hypercapnia was allowed as long as arterial pH was maintained ≥7.35. Specific ventilatory management has been previously described in Robba et al. (12). Invasive arterial blood pressure (ABP), heart rate (HR), and end-tidal carbon dioxide (ETCO_2_) were continuously measured. Using a Masimo root device with pulse CO–oximetry sensors connected to Rainbow devices, total Hb, perfusion index (Pi), and pleth variability index were non-invasively measured.

### Hemodynamic Management

PLR test was performed to assess fluid responsiveness during episodes of hypotension. With the patient seated at 45° in a head-up semirecumbent position (T0_PLR_), patient's upper body was then lowered to a horizontal position with legs passively raised at 45°, for 30–90 s. At the end of the procedure, before repositioning patient's legs, T1_PLR_ was defined (13–15). We considered a positive PLR test with 5% of ETCO_2_ increase in Delta ETCO_2_ from T0_PLR_ to T1_PLR_ (16, 17), as a surrogate of 10% increase in stroke volume (cardiac output monitor) (13). As this study is observational and did not change our practice, we did not have the possibility during the pandemic to use a more advanced hemodynamic tool in all our patients. Despite ETCO_2_ is not the criterion standard for PLR test evaluation, it has been previously used, and it demonstrated to be strongly associated with stroke volume changes, and therefore, it is now widely acceptable (13, 14, 17). According to the ETCO_2_ response to the PLR test as well as recommendation of the attending physician, patients received FC [crystalloids (4 mL/kg over 20 min)] (14, 15, 18) or NE infusion (central venous at a controlled rate using an infusion pump with an initial dose of 0.05 μg/kg per minute). NE infusion was then eventually started in PLR non-responding patients, or after FC, according to recommendation of attending physician if another episode of hypotension occurred. T0_FC_ and T0_NE_ were evaluated at the beginning of FC and NE infusion, whereas T1_FC_ and T1_NE_ were considered at the end of FC administration or at timepoints of 5 min after the beginning of NE, when the NE dosage was titrated (starting with a dose of 0.05 μg/kg per minute) to maintain an MAP >65 mm Hg. A complete hemodynamic assessment (HR, ABP, MAP, Hb, Spo_2_, Pi, and PVI) and neuromonitoring evaluation (TCD- and NIRS-derived indices) were obtained at timepoints T0 and T1 of FC and NE administration. No other interventions (such as mechanical ventilation settings changes, repositioning of the patient) were performed between T0 and T1.

### Neuromonitoring Data

During the second wave of the pandemic, we started to use neuromonitoring tools in all our patients as routine, at least in the early phases from ICU admission, as we noticed a high number of neurological complications (5).

### Cerebral Oxygenation

Masimo Root monitor® (USA) was used for the continuous measurement of rSo_2_ through bilateral sensors applied to the frontotemporal area. Different indices derived were obtained including (1) rSo_2_, which represents the total regional cerebral oxygen saturation value; (2) variation of O_2_Hbi, ΔO_2_Hbi, which represents the modifications of the oxygenated (arterial) component of the Hb of the total rSo_2_, whereas ΔHHbi defines the variation of the deoxygenated (venous) component of Hb of the total value of rSo_2_; (3) ΔcHbi, which is the sum of the values of ΔO_2_Hbi and ΔHHbi; and (4) ΔSpo_2_-rSo_2_ which represents the differences between the value of systemic and cerebral oxygenation. Final values were calculated as the mean between the right and left frontotemporal sensors.

### Calculation of Static CA

CA index was measured using TCD. Percentage change in estimated cerebrovascular resistance (CVR_e_) in relation to the change in ABP over the entire period of time needed for an MAP increase from baseline (T0) to the higher level (T1) was calculated as CVR_e_ = MAP/(cerebral blood flow velocity) (19). We calculated CA index as CA = (% ΔCVR_e_/% ΔMAP) × 100% as previously described (19). We expressed the CA index as a percentage of full autoregulatory capacity. A change in CVR, which would fully compensate for the drop in MAP, would yield a static CA of 100%, whereas no response of CVR, after ABP changes, would yield a static CA of 0%.

### nICP Assessment

Transcranial color duplex Doppler technique (Philips, Bothell, WA, USA) was performed with a low-frequency (2 MHz) echographic micro convex through a temporal window to assess bilateral middle cerebral arteries (MCAs). Systolic, diastolic, and mean flow velocity (FVs, FVd, and FVm, respectively) were obtained bilaterally from the MCA.

nICP was measured using two different formulas:

(1) FVd formula (nICPFVd) (20, 21):

$$\begin{array}{l} \text{nCPP}=\text{MA}{\text{P}}^{*}\left(\text{FVd}/\text{FVm}\right)+\;14, \end{array}$$

where nCPP is non-invasive cerebral perfusion pressure and then

$$\begin{array}{l} \text{nICP}=\text{MAP}-\text{nCPP}, \end{array}$$

(2) Pulsatility index (PI)–based nICP (nICPPI):

PI was measured according to the Gosling formula (22):

$$\begin{array}{l} \text{PI}=\left(\text{FVs}-\text{FVd}\right)/\text{Fm}, \end{array}$$

Estimation based on TCD-derived PI was based on the linear regression among known values of ICP and PI previously analyzed by Budohoski et al. (23):

$$\begin{array}{l} \text{nICPPI}=4.47^{*}\text{PI}+\;12.68 \end{array}$$

The final nICPPI was calculated using the mean of the right and left PI, whereas nICPFVd was calculated using the mean flow velocity of both MCAs.

### Statistical Analysis

No data on cerebral oxygenation after PLR test, FC, and NE are available in COVID-19 patients. Therefore, a formal sample size calculation was not feasible *a priori*. However, the achieved sample size was comparable to other physiologic studies in the field (24). The Shapiro–Wilk test was used to test the normality of the distribution of the results. Data are reported as median and interquartile range (IQR = 25th−75th percentiles), if not otherwise specified. Comparisons between different variables at T0 and T1 were performed by paired *t*-test, whereas non-normally distributed variables were compared by Wilcoxon signed rank test. One-way repeated-measures analysis of variance and Friedman test, followed by Bonferroni *post-hoc* test, were used for parametric and non-parametric data, respectively. Correlations between cerebral and systemic oxygenation were evaluated using Pearson or Spearman test. Correlations with repeated measurements were computed according to the Bland and Altman method (25). All statistical analyses were performed using SPSS 21® (IBM Corp., USA). *p* < 0.05 was considered statistically significant.

## Results

Baseline characteristics of the patients are presented in Table 1. The median age of the population was 62 years (IQR = 57–68.5 years), and 78% of the patients were male. Table 2 presents the hemodynamic and neuromonitoring parameters analyzed in the different subgroups [PLR (*n* = 23), FC (*n* = 22), and NE (*n* = 14)]. In all cases, PLR test was positive, and in 22 cases, FC was used as first-line treatment. In one patient, who presented with important fluid overload and respiratory failure, NE was started even with a positive PLR, based on the recommendation of ICU physician and patient's clinical conditions. After FC administration, despite a good initial response of MAP, 14 patients also required NE following another episode of hypotension (range between 0.05 and 1.5 μg/kg per minute).

**Table 1:** Characteristics of the patients included in the study.

**Characteristics of patients**	**All patients (*n* = 23)**
**Demographics**	
Gender, male, *n* (%) Age (years), median (IQR) BMI (kg/m^2^), median (IQR) PBW (kg), median (IQR)	18 (78.3) 62 (57–68.5) 26 (24.7–29) 70 (61–75)
**Comorbidities**	
Respiratory disease, *n* (%) Cardiovascular disease, *n* (%) Cancer, *n* (%) Moderate/severe liver disease, *n* (%) End-stage kidney injury, *n* (%) Hypertension, *n* (%) Diabetes mellitus, *n* (%)	3 (13.4) 5 (21.7) 0 (0) 1 (4.3) 0 (0) 13 (56.5) 3 (13)
**ICU characteristics at admission**	
SOFA score, median (IQR) Pao_2_/Fio_2_, median (IQR) PEEP, median (IQR) V_T_ (mL), median (IQR) Pplat,rs (cm H_2_O), median (IQR) Crs (mL/cm H_2_O), median (IQR) D-Dimer (ng/mL), median (IQR) C-reactive protein (mg/dL), median (IQR) Procalcitonin (ng/mL), median (IQR) Interleukin 6 (pg/dL), median (IQR) Creatinine (mg/dL), median (IQR) Heart rate (bpm), median (IQR) Mean arterial pressure (mm Hg), median (IQR)	5 (4–7) 81 (65–83) 11 (9–12) 434 (370–560) 26.5 (25–28) 24 (22–30) 1794 (1251–6252) 108.5 (60–135) 0.6 (0.18–1.76) 39 (31–83.3) 0.7 (0.6–1.1) 85 (72–97) 78 (73–85)
**ICU discharge characteristics**	
Dead, *n* (%) Alive, *n* (%)	13 (56.5) 10 (43.5)

*IQR, interquartile range; n, number; BMI, body mass index; PBW, predicted body weight; SOFA, Sequential Organ Failure Assessment; ICU, intensive care unit; Pao_2_/Fio_2_, arterial oxygen partial pressure/fraction of inspired oxygen; PEEP, positive end expiratory pressure; V_T_, tidal volume; Pplat,rs, respiratory system plateau pressure; Crs, respiratory system compliance*.

**Table 2:** Hemodynamic and neuromonitoring variables before (T0) and after (T1) passive leg raising test, fluid challenge, and norepinephrine.

	**Passive leg raising test (*****n*** **=** **23)**			**Fluid challenge (*****n*** **=** **22)**			**Norepinephrine (*****n*** **=** **14)**		
**Parameter**	**T0**	**T1**	***p*-Value**	**T0**	**T1**	***p*-Value**	**T0**	**T1**	***p*-Value**
**Hemodynamics**									
MAP (mm Hg)	63 (59–64.5)	69 (67.5–71.5)	<0.001^*^	61 (59–65)	69 (67–72)	<0.001^*^	63.5 (61–64)	69 (66–71)	<0.001^*^
HR (bpm)	76 (66.5–88.5)	77 (67–86)	0.822	75.5 (67–89)	75 (67–92)	0.910	76.5 (65–87)	78.5 (67–87)	0.239
Hb (g/dL)	7.9 (7.6–8.4)	8.2 (7.9–8.4)	0.132	7.7 (7.1–8.6)	8.5 (8.1–8.8)	0.434	8.3 (7.8–8.4)	8.8 (8.2–9)	0.432
PVI	20 (16–24)	15.5 (12–19)	<0.001^*^	20 (16–24)	16 (12–19)	<0.001^*^	19 (16–23)	19 (15–23)	0.324
Pi	3 (3–4)	4 (4–5)	<0.001^*^	3 (3–4)	4 (4–5)	<0.001^*^	3 (3–4)	4 (3–4)	0.046^*^
**Neuromonitoring**									
rSo_2_ (%)	52 (51–59.5)	57 (54–63.5)	<0.001^*^	55 (52–60)	58 (56–65)	<0.001^*^	54.5 (53–62)	61.5 (61–66)	0.001^*^
ΔcHbi	4.7 (3.6–6.6)	6 (5–8)	<0.001^*^	5.3 (3.1–7)	6.6 (4.5–8.4)	<0.001^*^	5.3 (4.3–8.1)	7.3 (5.4–10.5)	0.001^*^
ΔO_2_Hbi	3.8 (2.8–4.5)	4.1 (3.2–5)	<0.001^*^	3.5 (2.8–4.6)	3.9 (3.2–5.6)	<0.001^*^	3.9 (3.2–4.3)	5.7 (3.9–7.1)	0.001^*^
ΔHHbi	1.1 (0.8–1.9)	2.1 (1.8–2.9)	<0.001^*^	1.2 (0.4–2.1)	2.4 (1.4–3.1)	<0.001^*^	1.8 (0.7–2.9)	1.9 (0.9–3)	0.143
nICPPI (mm Hg)	18.6 (17.7–19.6)	19.3 (18.2–19.8)	0.009^*^	19.6 (14.8–21.1)	18.2 (16.4–19.1)	0.153	17.6 (16.5–20.3)	17.5 (16.5–19.9)	0.216
nICPFVd (mm Hg)	12.9 (8.5–18)	15 (10.5–19.7)	0.001^*^	10.9 (6.7–15.7)	14.4 (5.6 (21.1)	0.004^*^	10 (6.9–21.3)	12.2 (5.5–22.6)	0.022^*^
nCPP (mm Hg)	49.9 (42.1–53.8)	54.5 (49.3–59.6)	<0.001^*^	49.6 (44.8–61.1)	54.9 (50.7–65)	<0.001^*^	53.5 (41.2–60.5)	57.4 (45.3–62.5)	<0.001^*^
CVR (mm Hg/cm per second)	1.1 (1–1.2)	1.2 (1.1–1.4)	<0.001^*^	1.1 (1–1.2)	1.2 (1.1–1.3)	<0.001^*^	1.2 (1.1–1.2)	1.2 (1.2–1.3)	0.001^*^
**Others**									
ETCO_2_ (mm Hg)	43 (40–47)	46 (43–52.5)	0.001^*^	43 (38–51)	46.5 (44–50)	0.047^*^	43.5 (38–47)	45 (41–55)	0.001^*^
Spo_2_ (%)	90 (88–92)	91 (88–92)	0.854	90 (89–92)	91 (89–92)	0.357	89.5 (87–94)	89.5 (87–94)	0.317
DeltaSpo_2_-rSo_2_ (%)	36 (32–38.5)	32 (28–35.5)	<0.001^*^	35 (30–37)	32 (27–35)	<0.001^*^	33 (29–37)	26 (25–32)	0.001^*^

*Values are expressed as median and interquartile range if not otherwise specified. Hemodynamic and neuromonitoring variables before (T0) and after (T1) passive leg raising test, fluid challenge, and norepinephrine. Values are expressed as median and interquartile range if not otherwise specified. rSo_2_, cerebral oxygenation saturation; Delta O_2_Hbi (ΔO_2_Hbi), variation of the oxygenated component of the hemoglobin (Hb); Delta HHbi (ΔHHbi), variations of the deoxygenated component of Hb; Delta cHbi (ΔcHbi), sum of the values of ΔO_2_Hbi and ΔHHbi in the calculation of rSo_2_ value (ΔcHbi = ΔHHbi + ΔO_2_Hbi); ΔSpo_2_-rSo_2_, difference between the value of Spo_2_ and rSo_2_; N, number; MAP, mean arterial pressure; HR, heart rate; Pi, perfusion index; PVI, pleth variability index; ETCO_2_, end-tidal carbon dioxide; nCPP, non-invasive cerebral perfusion pressure; nICPPI, non-invasive intracranial pressure (ICP) based on pulsatility index; nICPFVd, non-invasive ICP based on flow velocity diastolic (FVd) formula; CVR, cerebrovascular resistance*.

* *p < 0.05*.

### Effect of PLR Test, FC, and NE on CA

**Figure 1:**
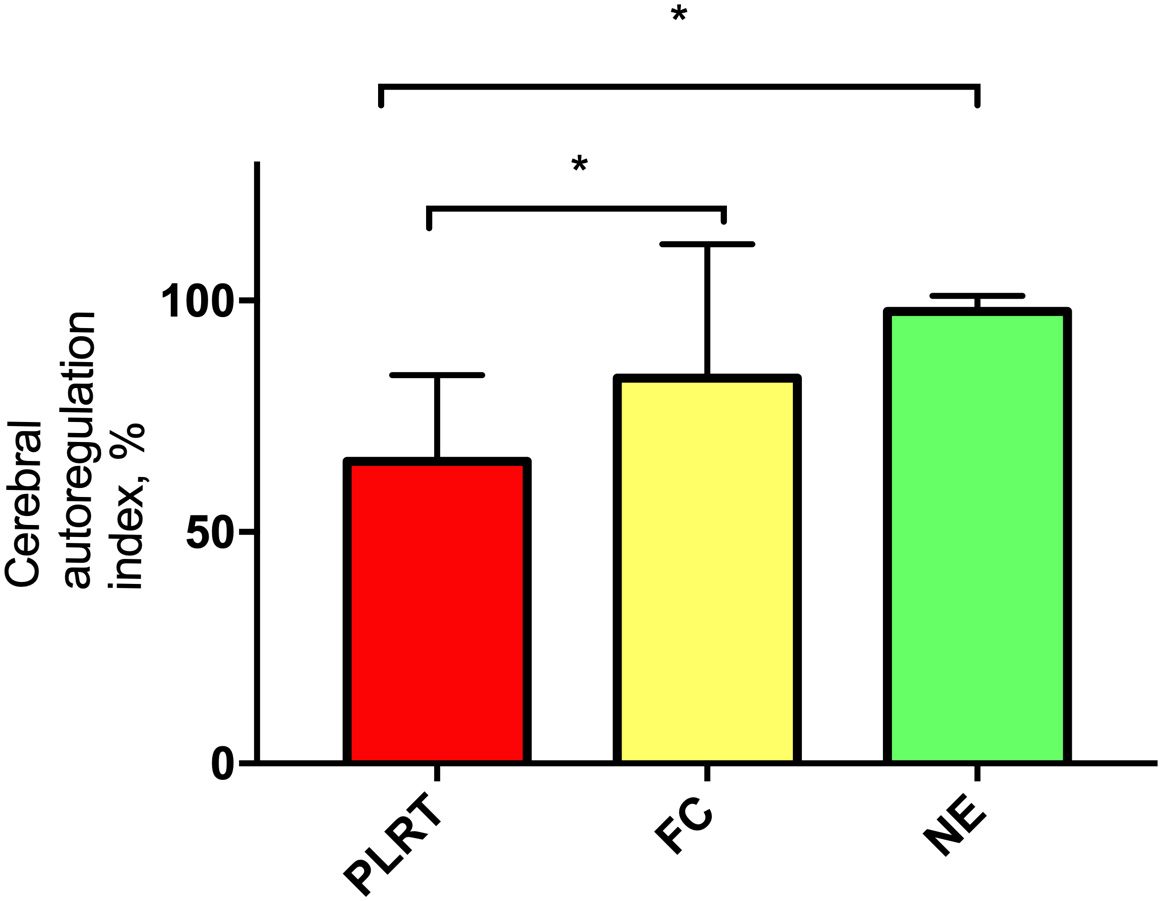
Percentage of cerebral autoregulation index. Data are expressed as mean, standard deviation. PLRT, passive leg raising test; FC, fluid challenge; NE, norepinephrine.

PLR test resulted in a CA index of 58% (44–76.3%), whereas FC and NE of 90.8% (74.2–100%) and 98% (96–100%), respectively. CA index did not differ between FC and NE (*p* = 0.169) (Figure 1). nICPPI (*p* = 0.542), nICPFVd (*p* = 0.529), and nCPP (*p* = 0.722) did not differ significantly between FC and NE (Supplementary Figures 1–3). NE yielded higher values of rSo_2_ compared to FC (Supplementary Figure 4) (NE vs. FC; *p* = 0.043).

### Effect of PLR Test, FC, and NE on Systemic Hemodynamics, Cerebral Oxygenation, and nICP

#### PLR Test

After PLR test, MAP was increased [63 (59–64.5) vs. 69 (67.5–71.5) mm Hg, *p* < 0.001] and rSo_2_ [52% (51–59.5%) vs. 57% (54–63.5%), *p* < 0.001], as well as nICPPI and nICPFVd [18.6 (17.7–19.6) vs. 19.3 (18.2–19.8) mm Hg, *p* = 0.009, and 12.9 (8.5–18) vs. 15 (10.5–19.7) mm Hg, *p* = 0.001, respectively (Table 2)].

#### Fluid Challenge

Fluid administration resulted in a significant increase in MAP [61 (59–65) vs. 69 (67–72) mm Hg, *p* < 0.001], rSo_2_ [55 (52–60) vs. 58 (56–65)%, *p* < 0.001], and nICPFVd [10.9 (6.7–15.7) vs. 14.4 (5.6- 21.1) mm Hg, *p* = 0.004] but not nICPPI [19.6 (14.8–21.1) vs. 18.2 (16.4–19.1) mm Hg, *p* = 0.153] (Table 2).

#### Norepinephrine

The administration of NE led to a significant increase in MAP [63.5 (61–64) vs. 69 (66–71) mm Hg, *p* < 0.001] and of rSo_2_ [54.5% (53–62%) vs. 61.5% (61–66%), *p* = 0.001] (Table 2), but only the arterial component improved [ΔO_2_Hbi = 3.9 (3.2–4.3) vs. 5.7 (3.9–7.1), *p* = 0.001]. nICPFVd slightly increased [10 (6.9–21.3) vs. 12.2 (5.5–22.6) mm Hg, *p* = 0.022], but not nICPPI [17.6 (16.5–20.3) vs. 17.5 (16.5–19.9) mm Hg, *p* = 0.216].

## Discussion

In the present study, we investigated the effects of PLR test, FC, and NE on cerebral physiology in mechanically ventilated patients with severe COVID-19 pneumonia. We found that (1) PLR test is associated with impaired CA; (2) FC and NE yield increased MAP, CPP, and rSo_2_. However, rSo_2_ is significantly higher after NE therapy compared to FC.

This is the first study exploring the effects of hemodynamic changes on cerebral hemodynamics in critically ill COVID-19 patients undergoing mechanical ventilation. This topic is of clinical importance, but not sufficiently highlighted in the literature. Patients with COVID-19 are at high risk of hemodynamic instability, because of sedation, mechanical ventilation, and eventually sepsis with direct negative effect on cardiac function, yielding cardiological complications (26–28). Maintenance of hemodynamic stability and the prompt treatment of hypotensive events are fundamental in this cohort of patients, to provide systemic organ perfusion and an appropriate CPP to the brain (29). In fact, COVID-19 patients are at risk of short- and long-term neurological complications (5, 8). The occurrence of stroke is increased in this population (30), and it appears to be greater in patients with COVID-19 when compared to those with influenza (1.6 vs. 0.2%, respectively, with odds ratio 7.6) (31). This higher incidence has been attributed to the increased incidence of both venous and arterial thromboembolism. In this context, it is well-known that hemodynamic instability and in particular hypotension and altered autoregulation are important risk factors for cerebral damage and secondary brain injury (6, 29, 32, 33). Although COVID-19 patients are not primarily brain-injured patients, recent evidence suggests that the cerebrovascular dynamics are impaired in this cohort of patients, with altered intracranial pressure and pupillometer indexes in most cases (8, 10, 34). Moreover, impaired CA is associated with poor outcome not only in brain-injured patients, but also in several other groups of patients, such as sepsis and cardiac arrest (6, 34, 35). PLR test is often used in the clinical practice as a test to assess the need for fluids and to help in the decision of starting fluid therapy or vasopressors; however, PLR test causes an intrinsic increase in intravascular volume, with shift of intravascular fluids from the legs to the abdominal compartment. This may increase intra-abdominal and intrathoracic pressures (36), yielding impaired cerebral hemodynamics and for this reason has been often discouraged in patients at risk of intracranial complications (37). There are few previous studies including a minority of ICU patients with brain injury who underwent PLR test [i.e., two of 34 patients in the study by Biais et al. (38), six of 71 patients in the study by Monnet et al. (39)], and no specific data on this subpopulation are available. The best hemodynamic strategy to optimize cerebral perfusion and autoregulation remains unclear. Fluid therapy is often used as first-line therapy in critically ill patients with hypotension (36), inducing a transient increase in cardiac preload consequent to extrinsic increase in intravascular volume, whereas vasopressors are started to improve MAP acting on vasomotor tone. The choice to use PLR test in these patients and treat them with fluids or vasopressors should depend on both lung and cerebral needs, which are often in conflict (40, 41). Our results suggest that PLR test may significantly decrease the cerebral autoregulatory system function of COVID-19 patients. PLR test can also increase ICP, suggesting that it may not be appropriate in patients at risk of cerebral complications. FC and NE were able to increase MAP and CPP, but NE seems to have a better effect on cerebral oxygenation and autoregulation, even though no differences in MAP and CPP between the three strategies were observed at T1.

The impact of vasopressors and FC on CA is not completely understood. Klein et al. (42) recently found in a cohort of 91 traumatic brain injury and 13 stroke patients that dynamic intracranial pressure–based measurements of cerebrovascular reactivity are not affected by NE. Similarly, Johnston et al. (43) suggested that CPP augmentation with NE, but not with dopamine, resulted in a significant reduction in arterial–venous oxygen difference (37 ± 11 vs. 33 ± 12 mL/L) and a significant increase in brain tissue oxygen (2.6 ± 1.1 vs. 3.0 ± 1.1 kPa).

The mechanism according to which NE—compared to fluids—could potentially better preserve autoregulation and cerebral oxygenation might be related to a greater effect of NE on the arterial component of rSo_2_, ΔO_2_Hbi, compared to FC. Furthermore, this could be related to specific characteristics of NE and the brain-blood barrier (BBB), which contains monoaminoxidase, thus allowing preserving cerebral vessels from its potential vasoconstrictor effects. Indeed, previous evidence confirms that NE has neuroprotective effects and improves CA (44) and oxygenation (43), leading to a significant increase in cerebral blood flow, in both conditions when BBB is intact or experimentally opened (45). No data are available regarding the effect of FC on CA so far, but potentially changes in plasma osmolarity and vascular content might have less protective effect on cerebral hemodynamics and BBB.

### Limitations

This study presents several limitations. First, the sample size of the patients included is small, especially considering each subgroup; however, no data are available in COVID-19 patients on this topic. Moreover, the number of patients in the current study is higher than that in similar studies on brain-injured patients (46). Second, the response to PLR test was defined according to changes of ETCO_2_, which is not the criterion standard (36, 47); the ETCO_2_ has been already used in patients with acute respiratory distress syndrome and NE infusion showing a better performance, as compared to systemic pressure (48). To the best of our knowledge, no data are available so far in COVID-19 patients regarding this use; however, we considered the performance of this surrogate good enough in a context of paucity of resources. Third, our results would have been strengthened by the availability of more specific data on physiological parameters including invasive neurologic, respiratory, and hemodynamic monitoring to assess the changes consequent to the application of the different hemodynamic strategies. In particular, the use of invasive ICP and oxygenation would have been of extreme utility, but no indications are available for their use in non-primarily brain-injured patients. Also, our population represents a specific subgroup with peculiar characteristics, and therefore, our results may not be applicable in other clinical settings. Fourth, PLR is a test, whereas FC and NE administration are two types of clinical interventions, and therefore, these are not comparable. The decision whether to avoid PLR test in patients at risk of cerebral complications should take into account patients' needs and clinical conditions, and further larger studies are warranted to clarify this issue. In addition, as some patients received both FC and NE, we cannot exclude a cumulative effect of these techniques. Finally, we evaluated the early effects of PLR test on static CA, and no information is provided from our results on a long-term effect of this technique.

## Conclusions

In mechanically ventilated severe COVID-19 patients, PLR test results in a reduction of cerebral autoregulatory function. PLR test, FC, and NE increased cerebral oxygenation, but NE seemed to have the major beneficial effect on cerebral oxygenation compared to FC. An individualized strategy aimed at assessing both the hemodynamic and cerebral needs is warranted in COVID-19 patients at high risk of neurological complications.

## Data Availability Statement

The raw data supporting the conclusions of this article will be made available by the authors, without undue reservation.

## Ethics Statement

The studies involving human participants were reviewed and approved by University of Genoa, Policlinico San Martino. The patients/participants provided their written informed consent to participate in this study.

## Author Contributions

CR: conceived of the study, designed the study, interpretation of the data, and drafted the manuscript. DB: acquired the data, interpretation of the data, and critical revision of the manuscript. AM and PP: interpretation of the data, critical revision of the manuscript, and final approval. LB, IB, NP, MCe, BM, XL, PR, MCz, DG, AV, and MB: critical revision of the manuscript and final approval. All authors read and approved the final manuscript.

## Conflict of Interest

The authors declare that the research was conducted in the absence of any commercial or financial relationships that could be construed as a potential conflict of interest.

## Abbreviations

ABP: arterial blood pressure
CA: cerebral autoregulation
COVID-19: coronavirus disease 2019
CPP: cerebral perfusion pressure
ETCO_2_: end-tidal carbon dioxide
FC: fluid challenge
Fio_2_: fraction of inspired oxygen
FVd: diastolic flow velocity
FVm: mean flow velocity
FVs: systolic flow velocity
Hb: hemoglobin
ICU: intensive care unit
MAP: mean arterial pressure
MCAs: middle cerebral arteries
nCPP: non-invasive cerebral perfusion pressure
NE: norepinephrine
nICP: non-invasive intracranial pressure
NIRS: near-infrared spectroscopy
Paco_2_: partial pressure of carbon dioxide
PEEP: positive end-expiratory pressure
Pi: perfusion index
PI: pulsatility index
PLR test: passive leg raising test
Pplat: plateau pressure
rSo_2_: cerebral oxygenation
Spo_2_: peripheral saturation of oxygen
TCD: transcranial Doppler.
